# Naturalistic psychedelic use and changes in depressive symptoms

**DOI:** 10.1016/j.jad.2025.119857

**Published:** 2025-07-09

**Authors:** Otto Simonsson, Peter S. Hendricks, Caroline M. Swords, Walter Osika, Simon B. Goldberg

**Affiliations:** a Center for Social Sustainability, Department of Neurobiology, Care Sciences and Society, Karolinska Institute, Solna, 171 65, Sweden; b Center for Healthy Minds, University of Wisconsin-Madison, Madison, WI 53703, USA; c Department of Psychiatry and Behavioral Neurobiology, University of Alabama at Birmingham, Birmingham, AL 35233, USA; d Department of Counseling Psychology, University of Wisconsin-Madison, Madison, WI 53706, USA; e Stockholm Health Care Services, Southern Stockholm Psychiatric District, Region Stockholm, Stockholm, Sweden

## Abstract

While growing evidence suggests that psychedelic-assisted therapy may have antidepressant effects in certain populations, little is known about the effects of psychedelic use on depressive symptoms in non-clinical, naturalistic settings. This observational cohort study included a large sample of US residents (18–50 years old) and longitudinally evaluated the relationships between naturalistic psychedelic use and changes in depressive symptoms. 21,990 participants completed the T1 survey and 12,345 completed the T2 survey (56.1 % retention). In total, 505 participants (i.e., 4.1 % of T2 survey completers) reported psychedelic use during the study period. The covariate-adjusted models showed a modest association between psychedelic use during the study period and increases in depressive symptoms (β = 0.12, *p* = .019). When disaggregated by context of use, psychedelic use in a ‘risk context’ (e.g., negative mindset prior to psychedelic experience, no psychological support present during psychedelic experience) was associated with moderate increases in depressive symptoms relative to no psychedelic use (β = 0.30, *p* < .001) and psychedelic use not in a ‘risk context’ (β = 0.27, *p* = .004). Notably, psychedelic use in a ‘risk context’ was strongly associated with having a more challenging psychedelic experience (β = 0.59, *p* < .001), which in turn was associated with modest increases in depressive symptoms (β = 0.16, *p* = .007) and accounted for the association between psychedelic use in a ‘risk context’ and increases in depressive symptoms. In conclusion, the findings suggest that naturalistic psychedelic use may not be generally therapeutic and may result in worsening depressive symptoms under certain circumstances. Future epidemiological research should further investigate factors associated with worsening depressive symptoms following naturalistic psychedelic use.

Growing evidence suggests that administration of psychedelics (e.g., psilocybin), paired with psychotherapy, may have antidepressant effects in certain populations. For example, in recent randomized trials, single-dose psilocybin-assisted therapy has been associated with rapid and sustained antidepressant effects in patients with depressive disorders (Goodwin et al., 2022; Raison et al., 2023). Other studies also indicate that psychedelic-assisted therapy has a relatively low risk of worsening depressive symptoms (Simonsson et al., 2023a) and other adverse events (Hinkle et al., 2024). However, these promising results have been accompanied by increases in naturalistic psychedelic use in countries such as the United States (US), especially among those with depressive symptoms or disorders (Rockhill et al., 2025; Walsh et al., 2024). It is therefore important to interrogate the effects of psychedelic use on depressive symptoms in contexts that more accurately reflect real-world scenarios of naturalistic psychedelic use, outside of controlled research and clinical settings.

The antidepressant effects reported in trials of psychedelic-assisted therapy have been partially replicated in longitudinal studies of naturalistic psychedelic use. For instance, in recent longitudinal observational cohort studies, naturalistic psychedelic use has been associated with reductions in depressive symptoms (Nayak et al., 2023; Nygart et al., 2022). It is important to note, however, that the longitudinal studies of naturalistic psychedelic use to date have various limitations that may introduce bias, including having many participants who self-report that they identify as advocates or experienced users of psychedelics (Haijen et al., 2018). These studies have typically also used recruitment materials that specifically target participants who plan to use psychedelics in the near future and have not included unexposed groups (i.e., participants who did not use psychedelics during the study period) with which to compare. Because of these limitations, it is possible that the results are influenced by expectancy, demand effects, or other biases, which could limit the generalizability of the results and the strength of conclusions that can be drawn about the possible impact of naturalistic psychedelic use on depressive symptoms. It is therefore difficult to make informed assessments of the potential risks and benefits of naturalistic psychedelic use that can guide public health efforts related to psychedelics.

Using a longitudinal observational cohort design, we conducted exploratory analyses to investigate associations between naturalistic psychedelic use and changes in depressive symptoms. Because previous research suggests that the context and the quality of the acute experience may be important predictors of psychedelic-related outcomes (Carhart-Harris et al., 2018), we conducted additional exploratory analyses to investigate associations between psychedelic use in a ‘risk context,’ the acute psychedelic experience, and changes in depressive symptoms.

## 1. Methods

### 1.1. Procedures

Using purposive sampling to maximize the amount of participants who would be likely to report psychedelic use during the study period, we targeted US residents between 18 and 50 years old, based on prior research (Simonsson et al., 2024). Participants were recruited from June–September in 2023 and in June 2024 through Prolific Academic, which is a participant recruitment platform for researchers with high-quality participation compared with other participant recruitment platforms (Douglas et al., 2023). The study description did not mention psychedelics to minimize the likelihood of selection bias. Participants provided informed consent, completed a baseline (T1) survey, and were invited to complete a follow-up (T2) survey two months later. This study was part of a larger study (Simonsson et al., 2025) and participants received a small payment for completion of T1 and T2 surveys. Study procedures were determined to be exempt from review by the Institutional Review Board at the University of Wisconsin-Madison.

### 1.2. Measures

At both T1 and T2, participants completed the 9-item self-reported Montgomery Åsberg Depression Rating Scale (Svanborg and Åsberg, 1994, 2001), which was developed to assess depression symptoms. At T1, participants were asked to report age, gender identity, educational attainment, degree of religiosity, political affiliation, past two-month use of psychedelics, and current or past diagnosis of any depressive disorders. At T2, participants were asked to report past two-month use of psychedelics, alcohol, nicotine products, cannabis products, MDMA, major stimulants, illicit narcotic analgesics or opioids, illicit benzodiazepines and barbiturates, inhalants, and other substances. Participants who reported past two-month psychedelic use at T2 were asked to recall their most intense psychedelic experience during that period, complete the Challenging Experiences Questionnaire (Barrett et al., 2016), and identify the dose size and context associated with the experience (Simonsson et al., 2023b). Based on previous research showing that three context-related variables (i.e., major life event prior to psychedelic experience, negative mindset prior to psychedelic experience, no psychological support present during psychedelic experience) may be associated with both a more challenging psychedelic experience and overall risk of harm (Simonsson et al., 2023b), we constructed a ‘risk-context’ variable with three levels (0 = no psychedelic use, 1 = psychedelic use and none of the three context-related risk factors, 2 = psychedelic use and at least one of the three context-related risk factors). Using three additional variables that may also be associated with a more challenging psychedelic experience (i.e., insufficient or inadequate preparation for the experience, disagreeable or uncomfortable physical environment, dose was too large; Simonsson et al., 2023b), we constructed an extended ‘risk-context’ variable with three levels (0 = no psychedelic use, 1 = psychedelic use and none of the six context-related risk factors, 2 = psychedelic use and at least one of the six context-related risk factors). We created composite ‘risk-context’ variables to capture overall contextual risk, which allowed us to avoid assumptions about the relative importance or interactions between individual factors. By not analyzing each potential risk factor individually, it also reduced the need for multiple statistical tests, thereby limiting inflation of Type I error rates.

### 1.3. Statistical analysis

We used linear regressions to investigate associations between the psychedelic-related variables (i.e., psychedelic use, psychedelic use in a ‘risk context’, severity of challenging psychedelic experience) and continuous outcome variables (i.e., changes in depressive symptoms, severity of challenging psychedelic experience). To improve interpretability of any significance changes, we also examined associations with minimal clinically important differences (i.e., standardized mean difference ≥ 0.24 or ≤ −0.24 for symptom worsening and improving, respectively; Cuijpers et al., 2014), which were used as outcome variables in logistic regressions. Similar to previous longitudinal observational cohort studies of naturalistic psychedelic use (Simonsson et al., 2024), we controlled for age, gender identity, educational attainment, degree of religiosity, political affiliation, past two-month use of psychedelics at T1, and past two-month use of alcohol, nicotine products, cannabis products, MDMA, major stimulants, illicit narcotic analgesics or opioids, illicit benzodiazepines and barbiturates, inhalants, and other substances at T2. The models that only included participants who reported psychedelic use during the study period also controlled for the dose size. We controlled for survey year in all analyses. All relevant models used the extended ‘risk context’ variable as outcome variable in sensitivity analyses. To produce standardized regression coefficients, we z-scored continuous variables using the ‘scale’ function in RStudio.

We used Multivariate Imputation by Chained Equations (Van Buuren and Groothuis-Oudshoorn, 2011) to handle missing data at T2 (no data were missing at T1) that were not missing-by-design. We imputed twenty data sets using random forest imputations and results were pooled with Rubin’s rules. A two-sided *p* < .05 was used as the significance threshold.

## 2. Results

### 2.1. Descriptive statistics

21,990 participants completed the T1 survey and 12,345 completed the T2 survey (56.1 % retention; Table 1). In total, 505 participants (i.e., 4.1 % of T2 survey completers) reported psychedelic use during the study period. There were differences between participants who reported psychedelic use during the study period and those who did not with regards to age, gender, and psychiatric history. Among those who reported psychedelic use during the study period, 36.4 % had their most intense psychedelic experience in the past two months in a ‘risk context’. Other statistics related to clinical symptomatology are presented in Table 2; additional sample characteristics have been reported elsewhere (Simonsson et al., 2025).

### 2.2. Regression models

The covariate-adjusted models showed a modest association between psychedelic use and increases in depressive symptoms (β = 0.12, *p* = .019; Table 3, Model 1). When disaggregated by context of use, psychedelic use in a ‘risk context’ was associated with moderate increases in depressive symptoms relative to no psychedelic use (β = 0.30, *p* < .001; Table 3, Model 2) and psychedelic use not in a ‘risk context’ (β = 0.27, *p* = .004; Table 3, Model 3). Significance tests and coefficient directions were largely unchanged in sensitivity analyses using complete (non-imputed) data (eTable 1 in Supplement). Among participants who reported psychedelic use during the study period, psychedelic use in a ‘risk context’ was strongly associated with having a more challenging psychedelic experience (β = 0.59, *p* < .001; Table 4, Model 1), which in turn was associated with modest increases in depressive symptoms (β = 0.16, *p* = .007). When modeled simultaneously, a more challenging psychedelic experience remained modestly associated with changes in depressive symptoms (β = 0.14, *p* = .031; Table 4, Model 4) while psychedelic use in a ‘risk context’ was no longer a significant predictor (β = 0.14, *p* = .224; Table 4, Model 4). The analyses of minimal clinically important differences are reported in eTables 2–4 in Supplement. Additional sensitivity analyses using the extended ‘risk context’ variable showed broadly the same results across all models.

## 3. Discussion

This study investigated associations between naturalistic psychedelic use and changes in depressive symptoms. The results showed that psychedelic use during the study period was associated with increases in depressive symptoms, but such increases were only observed for participants who reported psychedelic use in a ‘risk context’. Notably, psychedelic use in a ‘risk context’ was associated with having a more challenging psychedelic experience, which in turn was associated with increases in depressive symptoms and accounted for the association between psychedelic use in a ‘risk context’ and increased depressive symptoms.

The findings in this study on the associations between naturalistic psychedelic use and changes in depressive symptoms contrast with findings from previous longitudinal studies that found reductions in depressive symptoms (Nayak et al., 2023; Nygart et al., 2022), with discrepancies possibly attributed to differences in sample characteristics and contexts of naturalistic psychedelic use among participants. Other findings that stand out include the positive association between challenging psychedelic experience severity and worsening depressive symptoms, which contrasts with null findings in prior longitudinal studies (Nayak et al., 2023; Nygart et al., 2022). It is possible that the relationship between challenging psychedelic experience severity and mental health outcomes is nonlinear, with symptoms potentially worsening or improving at different stages following the psychedelic experience. It is also possible that the impact of a challenging psychedelic experience may depend on whether it is transient and resolved quickly or persists for a long time, paralleling how short-term versus chronic stress can lead to divergent long-term health outcomes (Dhabhar, 2018). These possibilities could explain contrasts in findings across studies and should be investigated further in future research.

While this study had substantial strengths (e.g., large sample, unexposed group with which to compare, no mention of psychedelics in recruitment materials), it also had important limitations. First, we used purposive sampling rather than representative sampling and recruited the sample from an online participant recruitment platform. These factors limit the generalizability of findings beyond the study sample. Second, we used multiple imputation to address the issue of missing data, but the study experienced a relatively high attrition rate (43.9 %), which may have introduced bias. Third, we collected limited data on the context of reported psychedelic use during the study period and other unmeasured contextual variables (e.g., intention for psychedelic use) may have provided additional insight into potential risk and protective factors. We also did not investigate interactions between individual factors, which could have added further nuance to our findings. Fourth, all data were self-reported, which introduces the possibility of recall bias, social desirability bias, or misreporting. Fifth, the analyses were exploratory, no corrections were applied for multiple comparisons, and the observational design precludes any definitive causal inferences. As such, the findings should be interpreted with caution. Future research should more comprehensively address these limitations and further investigate under what circumstances and for whom naturalistic psychedelic use may be associated with worsening depressive symptoms.

In conclusion, despite study limitations, findings suggest that naturalistic psychedelic use may not be generally therapeutic and may result in worsening depressive symptoms under certain circumstances. Future epidemiological research should further investigate factors associated with worsening depressive symptoms following naturalistic psychedelic use.

## Supplementary Material

Appendix

Appendix A. Supplementary data

Supplementary data to this article can be found online at https://doi.org/10.1016/j.jad.2025.119857.

## Figures and Tables

**Table 1:** Descriptive statistics^a^.

	No. (%)		
	Psychedelic use during study period		
	Yes (*n* = 505)	No (*n* = 11,840)	*P* value
Age, y			0.024
18–24	106 (21.0 %)	2068 (17.5 %)	
25–34	209 (41.4 %)	4747 (40.1 %)	
35–44	146 (28.9 %)	3546 (30.0 %)	
45–50	44 (8.7 %)	1479 (12.5 %)	
Gender identity			<0.001
Male	231 (45.7 %)	4210 (35.6 %)	
Female	248 (49.1 %)	7081 (59.8 %)	
Other	26 (5.2 %)	549 (4.6 %)	
Personal history of DD			<0.001
Yes	226 (44.8 %)	4220 (35.6 %)	
No	279 (55.3 %)	7620 (64.4 %)	
Psychedelic use in a ‘risk context’			
Yes	184 (36.4 %)	NA	
No	321 (63.6 %)	NA	

a This table shows complete (non-imputed) data on descriptive statistics of participants who completed both T1 and T2 surveys (*n* = 12,345). Percentages were rounded to the nearest 0.1 %; total percentages may not sum to exactly 100.0 %. Chi-square tests for categorical variables were used to examine differences between the two groups. DD = depressive disorders. NA = not applicable.

**Table 2:** Clinical statistics^a^.

	Mean (SD)		
	Psychedelic use during study period		
	Yes (n = 505)	No (n = 11,840)	P value
T1 MADRS-S total score	17.98 (9.85)	14.35 (9.77)	<0.001
T2 MADRS-S total score	18.58 (10.53)	14.36 (9.86)	<0.001
T1-to-T2 MCIDs			
Worsening	188 (37.2 %)	3510 (29.7 %)	<0.001
Improving	159 (31.5 %)	3599 (30.4 %)	0.603
T1 MADRS-S severity			<0.001
Absence of symptoms (0–6)	64 (12.7 %)	2943 (24.9 %)	
Mild depression (7–19)	217 (43.0 %)	5405 (45.7 %)	
Moderate depression (20–34)	198 (39.2 %)	3129 (26.4 %)	
Severe depression (35–54)	26 (5.2 %)	363 (3.1 %)	
T2 MADRS-S severity			<0.001
Absence of symptoms (0–6)	66 (13.1 %)	2977 (25.1 %)	
Mild depression (7–19)	210 (41.6 %)	5371 (45.4 %)	
Moderate depression (20–34)	196 (38.8 %)	3098 (26.2 %)	
Severe depression (35–54)	33 (6.5 %)	394 (3.3 %)	

a This table shows complete (non-imputed) data on clinical statistics of participants who completed both T1 and T2 surveys (n = 12,345). Mean (SD) presented for continuous variables. Number of cases (percentages) presented for categorical variables. Percentages were rounded to the nearest 0.1 %; total percentages may not sum to exactly 100.0 %. Chi-square tests were used to examine differences between the two groups for categorical variables, while independent *t*-tests were used to examine differences between the two groups for continuous variables. MCIDs = minimal clinically important differences (i.e., standardized mean difference ≥ 0.24 or ≤ −0.24 for symptom worsening and improving, respectively).

**Table 3:** Changes in symptoms predicted by psychedelic use^a^.

Changes in Depressive Symptoms			
Model	Predictor(s)	β (95 % CI)	P value
Model 1	Psychedelic Use	0.12 (0.02, 0.23)	0.019
Model 2	No Psychedelic Use	NA	NA
	Psychedelic Use Not in a ‘Risk Context’	0.03 (−0.09, 0.15)	0.618
	Psychedelic Use in a ‘Risk Context’	0.30 (0.14, 0.46)	<0.001
Model 3	Psychedelic Use Not in a ‘Risk Context’	NA	NA
	No Psychedelic Use	−0.03 (−0.15, 0.09)	0.618
	Psychedelic Use in a ‘Risk Context’	0.27 (0.09, 0.46)	0.004

a This table shows associations between psychedelic-related variables and changes in depressive symptoms using all participants (*n* = 21,990). Data missing at T2 were handled using multiple imputation. β = standardized coefficient; CI = confidence interval; NA = not applicable; all models controlled for age, gender identity, educational attainment, degree of religiosity, political affiliation, past two-month use of alcohol, nicotine products, cannabis products, MDMA, major stimulants, illicit narcotic analgesics/opioids, illicit benzodiazepines and barbiturates, inhalants, and other substances at T2, psychedelic use in the past two months at T1, and survey year.

**Table 4:** Acute experience and changes in symptoms predicted by psychedelic use^a^.

Severity of Challenging Psychedelic Experience			
Model	Predictor(s)	β (95 % CI)	P value
Model 1	Psychedelic Use Not in a ‘Risk Context’	NA	NA
	Psychedelic Use in a ‘Risk Context’	0.59 (0.44, 0.75)	<0.001
Changes in Depressive Symptoms			
Model	Predictor(s)	β (95 % CI)	P value
Model 2	Psychedelic Use Not in a ‘Risk Context’	NA	NA
	Psychedelic Use in a ‘Risk Context’	0.23 (0.01, 0.45)	0.044
Model 3	Severity of Challenging Psychedelic Experience	0.16 (0.04, 0.28)	0.007
Model 4	Psychedelic Use Not in a ‘Risk Context’	NA	NA
	Psychedelic Use in a ‘Risk Context’	0.14 (−0.09, 0.38)	0.224
	Severity of Challenging Psychedelic Experience	0.14 (0.01, 0.27)	0.031

a This table shows relationships between psychedelic use in a ‘risk context,’ the severity of a challenging psychedelic experience, and changes in depressive symptoms among participants who reported psychedelic use during the study period (n = 505). β = standardized coefficient; CI = confidence interval; NA = not applicable; all models controlled for age, gender identity, educational attainment, degree of religiosity, political affiliation, past two-month use of alcohol, nicotine products, cannabis products, MDMA, major stimulants, illicit narcotic analgesics/opioids, illicit benzodiazepines and barbiturates, inhalants, and other substances at T2, psychedelic use in the past two months at T1, the size of the dose used, and survey year. Model 4 included both predictors (Psychedelic Use in a ‘Risk Context’, Severity of Challenging Psychedelic Experience) in the model simultaneously.

## Data Availability

The fully anonymized non-imputed data for our primary analyses are publicly available at https://doi.org/10.6084/m9.figshare.29517875.v1
