# Supplementary material for: Naturalistic psychedelic use and changes in depressive symptoms
Source: J Affect Disord. Author manuscript; Available in PMC 2025 Jul 30. (PMC12308856; doi:10.1016/j.jad.2025.119857)
Supplement: Appendix [file NIHMS2099683-supplement-Appendix.docx]

**Supplement**

| **eTable 1.** Changes in Symptoms Predicted by Psychedelic Use (Non-Imputed Data) | | | |
| --- | --- | --- | --- |
| **Changes in Depressive Symptoms** | | | |
| **Model** | **Predictor(s)** | **β (95% CI)** | **P value** |
| Model 1 | Psychedelic Use | 0.11 (0.01, 0.22) | .033 |
|  |  |  |  |
| Model 2 | No Psychedelic Use | NA | NA |
|  | Psychedelic Use Not in a ‘Risk Context’ | 0.00 (-0.13, 0.12) | .956 |
|  | Psychedelic Use in a ‘Risk Context’ | 0.32 (0.16, 0.47) | <.001 |
|  |  |  |  |
| Model 3 | Psychedelic Use Not in a ‘Risk Context’ | NA | NA |
|  | No Psychedelic Use | 0.00 (-0.12, 0.13) | .956 |
|  | Psychedelic Use in a ‘Risk Context’ | 0.32 (0.14, 0.50) | <.001 |
| This table shows associations between psychedelic-related variables and changes in depressive symptoms using participants who completed both T1 and T2 surveys (n=12,345). β = standardized coefficient; CI = confidence interval; NA = not applicable; all models controlled for age, gender identity, educational attainment, degree of religiosity, political affiliation, past two-month use of alcohol, nicotine products, cannabis products, MDMA, major stimulants, illicit narcotic analgesics/opioids, illicit benzodiazepines and barbiturates, inhalants, and other substances at T2, psychedelic use in the past two months at T1, and survey year. | | | |

| **eTable 2.** Worsening or Improving Predicted by Psychedelic Use (Imputed Data) | | | |
| --- | --- | --- | --- |
| **Depressive Symptom Worsening (SMD ≥ 0.24)** | | | |
| **Model** | **Predictor(s)** | **aOR (95% CI)** | **P value** |
| Model 1 | Psychedelic Use | 1.21 (0.98, 1.50) | .071 |
|  |  |  |  |
| Model 2 | No Psychedelic Use | NA | NA |
|  | Psychedelic Use Not in a ‘Risk Context’ | 0.99 (0.76, 1.28) | .920 |
|  | Psychedelic Use in a ‘Risk Context’ | 1.74 (1.28, 2.38) | <.001 |
|  |  |  |  |
| Model 3 | Psychedelic Use Not in a ‘Risk Context’ | NA | NA |
|  | No Psychedelic Use | 1.01 (0.78, 1.32) | .920 |
|  | Psychedelic Use in a ‘Risk Context’ | 1.77 (1.21, 2.58) | .003 |
| **Depressive Symptom Improving (SMD ≤ -0.24)** | | | |
| **Model** | **Predictor(s)** | **aOR (95% CI)** | **P value** |
| Model 1 | Psychedelic Use | 0.80 (0.64, 1.00) | .052 |
|  |  |  |  |
| Model 2 | No Psychedelic Use | NA | NA |
|  | Psychedelic Use Not in a ‘Risk Context’ | 0.81 (0.62, 1.05) | .107 |
|  | Psychedelic Use in a ‘Risk Context’ | 0.77 (0.55, 1.08) | .125 |
|  |  |  |  |
| Model 3 | Psychedelic Use Not in a ‘Risk Context’ | NA | NA |
|  | No Psychedelic Use | 1.24 (0.95, 1.62) | .107 |
|  | Psychedelic Use in a ‘Risk Context’ | 0.95 (0.64, 1.42) | .817 |
| This table shows associations between psychedelic-related variables and depressive symptom worsening or improving using all participants (n=21,990). Data missing at T2 were handled using multiple imputation. SMD = standardized mean difference; aOR = adjusted odds ration; CI = confidence interval; NA = not applicable; all models controlled for age, gender identity, educational attainment, degree of religiosity, political affiliation, past two-month use of alcohol, nicotine products, cannabis products, MDMA, major stimulants, illicit narcotic analgesics/opioids, illicit benzodiazepines and barbiturates, inhalants, and other substances at T2, psychedelic use in the past two months at T1, and survey year. | | | |

| **eTable 3.** Worsening or Improving Predicted by Psychedelic Use (Non-Imputed Data) | | | |
| --- | --- | --- | --- |
| **Depressive Symptom Worsening (SMD ≥ 0.24)** | | | |
| **Model** | **Predictor(s)** | **aOR (95% CI)** | **P value** |
| Model 1 | Psychedelic Use | 1.29 (1.03, 1.61) | .024 |
|  |  |  |  |
| Model 2 | No Psychedelic Use | NA | NA |
|  | Psychedelic Use Not in a ‘Risk Context’ | 1.03 (0.78, 1.34) | .849 |
|  | Psychedelic Use in a ‘Risk Context’ | 1.85 (1.35, 2.55) | <.001 |
|  |  |  |  |
| Model 3 | Psychedelic Use Not in a ‘Risk Context’ | NA | NA |
|  | No Psychedelic Use | 0.97 (0.75, 1.28) | .849 |
|  | Psychedelic Use in a ‘Risk Context’ | 1.81 (1.24, 2.64) | .002 |
| **Depressive Symptom Improving (SMD ≤ -0.24)** | | | |
| **Model** | **Predictor(s)** | **aOR (95% CI)** | **P value** |
| Model 1 | Psychedelic Use | 0.87 (0.69, 1.09) | .226 |
|  |  |  |  |
| Model 2 | No Psychedelic Use | NA | NA |
|  | Psychedelic Use Not in a ‘Risk Context’ | 0.89 (0.68, 1.16) | .393 |
|  | Psychedelic Use in a ‘Risk Context’ | 0.83 (0.59, 1.16) | .292 |
|  |  |  |  |
| Model 3 | Psychedelic Use Not in a ‘Risk Context’ | NA | NA |
|  | No Psychedelic Use | 1.13 (0.86, 1.48) | .393 |
|  | Psychedelic Use in a ‘Risk Context’ | 0.94 (0.63, 1.39) | .744 |
| This table shows associations between psychedelic-related variables and depressive symptom worsening or improving using participants who completed both T1 and T2 surveys (n=12,345). SMD = standardized mean difference; aOR = adjusted odds ratio; CI = confidence interval; NA = not applicable; all models controlled for age, gender identity, educational attainment, degree of religiosity, political affiliation, past two-month use of alcohol, nicotine products, cannabis products, MDMA, major stimulants, illicit narcotic analgesics/opioids, illicit benzodiazepines and barbiturates, inhalants, and other substances at T2, psychedelic use in the past two months at T1, and survey year. | | | |

| **eTable 4.** Worsening or Improving Predicted by Psychedelic Use (Psychedelic Users Only) | | | |
| --- | --- | --- | --- |
| **Depressive Symptom Worsening (SMD ≥ 0.24)** | | | |
| **Model** | **Predictor(s)** | **aOR (95% CI)** | **P value** |
| Model 1 | Psychedelic Use Not in a ‘Risk Context’ | NA | NA |
|  | Psychedelic Use in a ‘Risk Context’ | 1.67 (1.11, 2.52) | .013 |
|  |  |  |  |
| Model 2 | Severity of Challenging Psychedelic Experience | 1.26 (1.01, 1.57) | .044 |
|  |  |  |  |
| Model 3 | Psychedelic Use Not in a ‘Risk Context’ | NA | NA |
|  | Psychedelic Use in a ‘Risk Context’ | 1.53 (0.99, 2.35) | .052 |
|  | Severity of Challenging Psychedelic Experience | 1.17 (0.92, 1.48) | .197 |
| **Depressive Symptom Improving (SMD ≤ -0.24)** | | | |
| **Model** | **Predictor(s)** | **aOR (95% CI)** | **P value** |
| Model 1 | Psychedelic Use Not in a ‘Risk Context’ | NA | NA |
|  | Psychedelic Use in a ‘Risk Context’ | 0.97 (0.63, 1.48) | .873 |
|  |  |  |  |
| Model 2 | Severity of Challenging Psychedelic Experience | 0.75 (0.58, 0.96) | .026 |
|  |  |  |  |
| Model 3 | Psychedelic Use Not in a ‘Risk Context’ | NA | NA |
|  | Psychedelic Use in a ‘Risk Context’ | 1.16 (0.73, 1.83) | .522 |
|  | Severity of Challenging Psychedelic Experience | 0.73 (0.56, 0.95) | .021 |
| This table shows relationships between psychedelic use in a ‘risk context,’ the severity of a challenging psychedelic experience, and depressive symptom worsening or improving among participants who reported psychedelic use during the study period (n=505). SMD = standardized mean difference; aOR = adjusted odds ratio; CI = confidence interval; NA = not applicable; all models controlled for age, gender identity, educational attainment, degree of religiosity, political affiliation, past two-month use of alcohol, nicotine products, cannabis products, MDMA, major stimulants, illicit narcotic analgesics/opioids, illicit benzodiazepines and barbiturates, inhalants, and other substances at T2, psychedelic use in the past two months at T1, the size of the dose used, and survey year. Model 3 included both predictors (Psychedelic Use in a ‘Risk Context’, Severity of Challenging Psychedelic Experience) in the model simultaneously. | | | |
